# Arabic translation, cross cultural adaptation, and validation of Foot Health Status Questionnaire among Saudi individuals with plantar fasciitis

**DOI:** 10.1186/s13018-023-04202-9

**Published:** 2023-10-04

**Authors:** Sulaiman Alshammari, Mohammed Abdulsalam M. Alshwieer, Saad Salem Dammas, Abdulaziz Mohammed Alrasheed, Mohammed Ali Alasmari, Mansour Mohammed Abdullah Alahmari, Alwaleed Khalaf Alazmi

**Affiliations:** 1 Department of Family and Community Medicine, College of Medicine, King Saud University Medical City, Riyadh, Saudi Arabia; 2 College of Medicine, King Saud University, Riyadh, Saudi Arabia

**Keywords:** Foot Health Status Questionnaire, FHSQ, Cross-cultural adaptation, Arabic, Quality of life

## Abstract

**Background:**

Measuring quality of life (QoL) plays an essential role in enabling meaningful cross-cultural comparisons. The Foot Health Status Questionnaire (FHSQ) is a valid tool for assessing both foot-specific and general health-related quality of life (HrQoL), making it suitable for evaluating Plantar Fasciitis (PF) patients.

**Methodology:**

The aim of this study is to translate the FHSQ into Arabic following methodological assessments of the translation procedure. The translation was done using forward and back translation. A pre-test questionnaire was distributed among 50 patients, resulting in the final FHSQ-Ar version, which then underwent various psychometric evaluations among 87 persons with PF, including internal consistency, dimensionality, reliability, interpretability, and construct validity against the 100-mm Visual Analogue Scale (VAS).

**Results:**

Internal consistency was adequate, ranging from 0.70 to 0.92. Reliability values ranged from 0.69 to 0.80, with a poor standard error of measurement (individual) but an acceptable standard error of measurement (group). Two domains exhibited floor effects, while one domain showed a ceiling effect. Regarding validity, three out of four hypothesized correlations with VAS scores were confirmed. Factor analysis revealed four dimensions, and confirmatory factor analysis demonstrated good fit (comparative fit index = 0.98, standardized root mean square = 0.06).

**Conclusion:**

The psychometric properties of the FHSQ-Ar were satisfactory. Further validation for other diseases may be warranted.

**Supplementary Information:**

The online version contains supplementary material available at 10.1186/s13018-023-04202-9.

## Introduction

Foot and heel pain is one of the most prevalent disorders of the body's musculoskeletal system [1, 2]. Although there is no specific prevalence in Arab region, foot pain is estimated to range globally from 9 to 36% in adults of the general population, with a pooled prevalence of 24%. One in four individuals will encounter foot pain at some point in their lives, and up to four out of ten runners will complain of foot-related pain [3–7]. It is the third most common site of self-reported pain, following knee and wrist pain, among those older than 55 years old [8].

Foot pain is highly prevalent and known to cause locomotor disability, impaired balance, an increased risk of falling, worse functional activities of daily living, and significant impacts on daily living tasks [5, 9–11]. Given both the ubiquitous occurrence of musculoskeletal issues in the foot and ankle across populations as well as their consequential effects, it is imperative to properly gauge the impact of foot pain on individuals and communities for effective healthcare service provision. However, there remains a lack of research investigating the burden of foot disabilities specifically among Arabic and Persian-speaking populations [12], despite the fact that these conditions are so common. Implementing valid patient-reported outcome measures translated into local languages could help enhance the evaluation and treatment of foot issues while providing valuable insights into disparities between diverse societies [9].

Many patient-reported outcome measures exist for the foot and ankle, measuring variable functions such as pain, disability, and general health. The Foot Health Status Questionnaire (FHSQ) is a comprehensive, self-administered questionnaire that assesses foot-specific and generic health-related quality of life (HrQoL). The foot-specific section is measured over four domains: Pain, Function, Footwear, and General Foot Health [13]. The FHSQ has been shown to have excellent reliability, validity, and responsiveness in many populations, especially in patients with plantar fasciitis (PF) [14]. he FHSQ questionnaire has been adapted and translated into many languages [9]. However, there are currently no validated Arabic versions of the FHSQ available. This limits the ability of researchers and healthcare professionals to assess foot health in Arabic-speaking populations.

The purpose of quality of life measures is to provide assessments of individuals or populations that are more precise and comparable to facilitate meaningful comparisons [15]. Consequently, venturing into cross-cultural adaptation can unlock valuable insights into a particular inquiry, or uncover disparities between diverse societies. To accomplish either of these objectives, researchers must have access to the same questionnaire translated into various languages. If a translated questionnaire already exists, it is prudent for researchers to adapt it, utilizing a validated questionnaire rather than creating a new one [16, 18]. This approach is called the cross-cultural adaptation process, and yields measurements that are deemed equivalent.

PF is one of the most common foot problems. It causes pain in the plantar fascia, a thick band of tissue that runs along the bottom of the foot. The plantar fascia originates at the heel bone (calcaneus) and inserts into the heads of the metatarsal bones. PF can impair foot function, limit physical activity, and reduce well-being [19]. The FHSQ Provides a valuable tool for evaluating foot health in Arabic-speaking populations.

This research aims to translate and culturally adapt the FHSQ into Arabic and investigate face and construct validity in individuals with PF.

## Methodology

### Design and ethical consideration

This study was approved by the Institutional Review Board (IRB) of King Saud University (project number E-23-7648). Prior to translating the questionnaire into Arabic, we obtained permission from the original questionnaire developers in February 2023 [13]. Recommended guidelines were followed for the transcultural adaptation of instruments using a translation-back translation process [17, 18]. All participants provided their informed consent in the first section before completing the questionnaire.

### Procedure

(i) *Translation*: The instrument (original FHSQ) underwent forward translation into the Arabic language by two independent bilingual translators whose Arabic is their native language with a bicultural background; the first translator is a healthcare professional, while the second translator lacks knowledge of medical terminology. Emphasis was placed on capturing the original concept rather than a literal interpretation; this approach yielded two translated versions (FHSQ-ArA1) and (FHSQ-ArA2).
(ii) *Comparison of the two Arabic-translated versions*: A third bicultural translator then compared the two translations and discussed any discrepancies with the initial two translators and the research team. During this step, a disagreement arose regarding translating “how often” in questions 2, 3, and 4. There are two equivalent translations in Arabic—"how many times" and "how usual". Ultimately, the translation aligning more closely with "How usual" was deemed appropriate and selected. This process culminated in the creation of a preliminary Arabic version of the FHSQ, labeled (FHSQ-ArA12).
(iii) *Blind back-translation*: Another team comprising two bilingual experts, who were unaware of the original version, matching the characteristics of the forward translators, engaged in blind back-translation of the preliminary (FHSQ-ArA12) into English. This step yielded two back-translated versions (FHSQ-EnA1) and (FHSQ-EnA2).
(iv) *Comparison of the two English back-translated versions and committee analysis*: In this step, the research committee, consisting of a methodologist and four bilingual translators, participated in forward and back-translation, and the research team scrutinized the instructions, response format, and items of the two back-translated versions with the original FHSQ. The comparison also included aspects of meaning similarity and the grammatical structure of sentences. During this step, one item had been corrected: questions 5 and 6, which appeared similar in terms of performing work. To address this, A semantic equivalence that can differentiate between difficulty caused by foot pain and limitation caused by foot pain was adopted. Finally, the research committee incorporated these changes into the preliminary Arabic version of the FHSQ yielding (FHSQ-ArA13).
(v) *Pre-test*: Following guidelines, a suggested 10–40 participants from the target population were required to conduct a pre-test study. [17, 18] The preliminary Arabic FHSQ questionnaire (FHSQ-ArA13). was filled out as an online questionnaire with instructions and informed consent on the first page. An initial 30 patients with variable foot and ankle conditions from the outpatient clinic at King Khalid University Hospital (KKUH) had filled out this online-based questionnaire. The participants were asked to indicate the duration required to complete the questionnaire. Also, they were asked to evaluate the instructions and items on the scale using a binary rating system of clear or unclear. In the event that any participant rates the instructions, response format, or any item of the instrument as unclear, they are requested to provide suggestions to rephrase the statements to make the language more clear. An inter-rater agreement of at least 80% was deemed acceptable [17]. During this phase, except for nine patient complaints about the Likert scales, no items were identified as unclear. The Likert scales were subsequently revised and corrected, as detailed in Table 5. The revised version was then administered to an additional 20 patients, making the total number of participants 50 with varying foot conditions. No instances of unclear ratings were recorded. With around 10 min as a mean to complete the questionnaire. With this step, the final version was released (FHSQ-Ar) as shown in Table 5.

### Sample size and population

A dataset of patients with PF was extracted from the E-Sihi system between 2016 and February 2023. 350 patients were selected using systematic random sampling; none of them participated in the pilot study. Participants received the online survey using Google Forms and WhatsApp or SMS messages. The expected response rate was 25–30% [20].

### Outcome measures

The first section of the distributed questionnaire contained instructions to instructions to fill it out, informed consent, The second section confirmed whether the patients still had the disease by defining.

PF pain as "painful heel syndrome with localized pain at the heel of the foot, worst in the morning or after resting for a long time." Participants were then asked about the severity using the VAS 100 mm score and the exact location of their pain. Demographic data was obtained at the same section, as shown in Table 6. The inclusion criteria for the study were as follows: Saudis, Arabic native speakers, and PF patients have heel pain that is worse in the morning or after resting for a long time. Patients who are illiterate, have extreme ages > 65 or < 18 years old, have a history of previous foot or ankle trauma or surgery, or have complained of another foot disease were excluded. The third and fourth sections contained foot-specific and generic FHSQ questions. respectively. No missing data in the FHSQ was accepted since filling out each item was mandatory.

Participants who filled out the VAS and FHSQ scores along with pre-prepared demographic data were asked to refill the questionnaire after 1 week to appreciate the test–retest reliability, reduce participants' ability to remember their previous responses, and present a more accurate representation of the extent of score fluctuations [21].

The FHSQ is divided into two sections, further detailed in eight domains. The first section includes four domains that evaluate foot health: Foot Pain, Foot Function, Footwear, and General Foot Health. The second four domains evaluate generic HrQoL: General Health, Physical Activity, Social Capacity and Vigor. The latter four domains in the FHSQ were derived from the validated Arabic SF-36 questionnaire and do not require any further cross-cultural adaptation [22].

The Visual Analogue Scale (VAS) was initially introduced in 1921 by Hayes and Patterson as a pain rating scale. Since then, it has been widely employed in both epidemiological and clinical research to assess the intensity or frequency of diverse symptoms. The basic VAS consists of a straight horizontal line with a fixed length, typically 100 mm. The two ends of the line represent the extreme boundaries of the parameter being measured, such as symptoms, pain, or health, with the left end denoting the worst and the right end denoting the best [23]. For this research, we included an image displaying a horizontal VAS 0–100 mm score, followed by a field for a brief answer. We then requested that the participants provide the severity of their pain over the past week in numerical form, using the image above as a reference.

### Statistical analysis

In our research, we used internal consistency, intraclass correlation coefficients, and measurement error with the smallest detectable change to check the reliability of the questionnaire.

(i) Internal consistency: Cronbach’s alpha was used as a measurement of the internal consistency of the Arabic FHSQ. Anything between 0.7 and 0.95 was considered good [24, 25].
(ii) Dimensionality using confirmatory factor analysis (CFA) was conducted after fulfilling the minimum of 5 participants per item appropriate to be conducted [26]. A first-order factor analysis was undertaken using principal components analysis, and factors were identified using a scree plot. Data for factor analysis was utilized through the RStudio Lavaan package [27]. The correlation matrix of factors was computed to provide direction for developing hypotheses during the second stage of confirmatory factor analysis.
(iii) The test–retest reliability of the (FHSQ-Ar) was estimated using intraclass correlation coefficients (ICC). Participants who reported changes in their complaints were not included in the test–retest analysis. The target was to have at least 30 participants complete the questionnaire again, and ultimately, 41 participants refilled the questionnaire for the retest estimation [28, 29]. ICC estimates and their corresponding 95% confidence intervals were computed using the absolute-agreement, 2-way mixed-effects model. An ICC greater than 0.7 was considered as good [25].
  To determine the standard error of the measurement (SEm); the standard deviation (SD) and the reliability score obtained from the test–retest calculation were utilized, where SEm = SD/square root of reliability of the specific item. The Smallest Detectable Change (SDC_individual_) was computed as SEM × 1.96 × √2. (SDC_group_) was carried out by dividing the (SDC_individual_)/√n [25].
(iv) Interpretability: Floor and ceiling effects were evaluated by determining whether more than 15% of the participants attained their answers on the boundaries of each item [25]. If so, these effects were deemed to be present.
(v) Construct validity: To evaluate the construct validity of FHSQ-Ar (measured on a 0–100 scale for each domain, from worst QoL to best QoL, respectively), Spearman’s correlation coefficient was used to analyze the relationship between each domain score and the mean heel pain in the past week (measured on a 0–100 mmVAS scale, no pain/no disability to most severe pain and disability). The spearman’s correlation coefficient is particularly useful for analyzing ordinal variables, and/or non-normally distributed variables [30]. A fair negative correlation was hypothesized between heel pain intensity and domain scores for ‘Pain’, ‘Function’, and ‘General Foot Health’. In contrast, a poor negative correlation was hypothesized between heel pain intensity and the Footwear domain score. The Footwear domain measures a construct thought to be independent from heel pain intensity based on the original instrument [13].

Values of r close to 0, either between 0 and 0.25 or between 0 and  − 0.25, generally suggest that there is little or no correlation between the variables. Values of r between 0.25 and 0.50 or between  − 0.25 and  − 0.50 indicate a poor correlation between the variables. *r* values in the range of 0.50 to 0.75 or  − 0.50 to  − 0.75 point to a moderate to reasonably good correlation between the variables. Finally, *r* values between 0.75 and 1 or  − 0.75 and  − 1 indicate a very strong to excellent correlation between the variables. [30, 31] When a minimum of 75% of the a priori hypotheses are verified, the instrument has an adequate level of construct validity [25].

After obtaining responses, FHSQ scores were administered into the Foot Health Status Questionnaire Data Analysis Software Version 1.04 to estimate an accurate measurement of the scales. SPSS version 26.0 (IBM Corp., Armonk, NY) was used for analyzing the data. Mean, standard deviation, median, and interquartile range (IQR) were utilized to describe the quantitative variables, while frequencies and percentages were used for the categorical variables. Evaluation of measurement properties, including reliability, dimensionality, construct validity, and interpretability, was performed following the method described above. Factor analysis was conducted using the Lavaan package (v0.6-7; Rosseel, 2012).

## Results

This study was conducted utilizing the translation and back-translation methods. The questionnaire was distributed to 300 patients diagnosed with PF in the E-Sihi system. Out of the 96 respondents (32% response rate), 87 participants (90.6%) were included in the analysis after applying the inclusion and exclusion criteria.

**Fig. 1:**
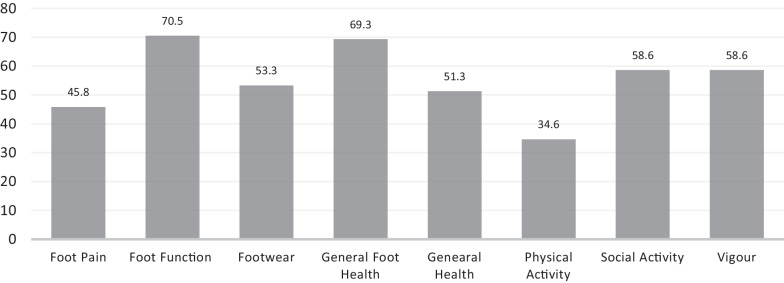
Mean scores of the Arabic Foot Health Status Questionnaire (FHSQ-Ar) domain among Plantar fasciitis patients

The participants mean age (± SD) at baseline was 48.6 (± 10.1), with the majority (68.6%) being females. Nearly 79.3% of the total population was married. Along with that, 78.5% had an educational level beyond high school. Over half of the participants (55.2%) didn’t engage in any form of exercise, and about 46.0% of all participants were either retired or unemployed. Further details and associated comorbidities are outlined in Table 6. A total of 38 participants were enrolled in test–retest analysis for reliability tests. Figure 1 outlines the mean FHSQ scores among this paper’s population.

### Reliability

The present study investigated the internal consistency, test–retest reliability, standard error of measurement, and smallest detectable difference (both individual and group) of the foot-specific FHSQ domains of the Arabic questionnaire.

The results, as outlined in Table 1 demonstrate that the internal consistency of all domains is estimated to have an excellent Cronbach's alpha ranging between (0.91) and (0.92), with the exception of the Footwear domain, which is estimated to have a good internal consistency (0.70).

**Table 1:** Item Internal consistency, Factor loading, item-scale correlation and reliability (N = 87)

		Internal consistency Cronbach’s alpha (CI)	Factor loading	Item-subscale correlation	Test–retest reliability (*n* = 41)
*Foot pain domain*					
Q1	ما مستوى الألم خلال الأسبوع الماضي؟	0.78	0.81	0.73	
Q2	هل تشعر عادةً بآلام القدم؟	0.56	0.86	0.83	
Q3	هل تشعر عادةً بآلام خفيفة متواصلة في قدمك؟	0.64	0.84	0.79	
Q4	هل تشعر عادةً بألم حاد في قدمك؟	0.76	0.90	0.84	
	Scale summary	0.91 (0.87; 0.94)			0.76 (0.59; 0.87)
*Foot Function domain*					
Q5	هل سببت لك قدمك صعوبات في أداء عملك أو نشاطاتك ؟	0.75	0.93	0.85	
Q6	هل حدّت قدمك من أدائك لأعمال اعتدت القيام بها ؟	0.70	0.87	0.82	
Q7	إلى أي درجة حدّت صحة قدمك من مشيك؟	0.81	0.85	0.82	
Q8	إلى أي درجة حدّت صحة قدمك من استخدامك السلالم للصعود؟	0.66	0.76	0.74	
	Scale summary	0.92 (0.88; 0.94)			0.75 (0.58; 0.86)
*Footwear Domain*					
Q10	من الصعب العثور على حذاء لا يؤذي قدمي	0.46	0.91	0.62	
Q11	من الصعب العثور على حذاء يناسب قدمي	0.70	0.74	0.59	
Q12	أنا مقيد في عدد الأحذية التي يمكنني ارتدائها	0.53	0.40	0.36	
	Scale summary	0.70 (0.57; 0.80)			0.69 (0.48; 0.82)
*General Foot Health Domain*					
Q9	ما تقيمك لصحة قدمك بصورة عامة؟	0.79	0.99	0.84	
Q13	بشكل عام، ما حال قدمك حاليًا؟	0.87	0.86	0.84	
	Scale summary	0.91 (0.87; 0.94)			0.80 (0.64; 0.89)

*CI* Confidence interval

Table 1 also presents the findings of the factor analysis performed on the preliminary sample size of 87 participants. We utilized a first-order factor analysis, implemented through principal components analysis (PCA), and used a scree plot for factor identification. With direct oblimin rotation in the PCA, 79% of the total variance was explained by four factors. These four factors contributed to the following percentages: 52.6, 13.6, 7.7, and 6.0%, respectively. The comparative fit index (CFI) was 0.98 with a standardized root mean square of 0.06. The Total scores of each of the four domains in a factor correlation matrix are shown in Table 2. The scores for all four subscales are presented in a factor correlation matrix. As anticipated, foot pain and function domains showed the highest correlation, indicating distinct yet complementary aspects of foot health. The GFH domain also showed a high correlation with both previous two domains. The Footwear domain didn’t show any correlation since it measures a distinctive entity.

**Table 2:** Factor correlation matrix for sub-scale total scores

	Pain	Function	Footwear	GFH
Pain domain	1.00			
Function domain	0.690	1.00		
Footwear domain	0.266	0.274	1.00	
GFH domain	0.646	0.689	0.131	1.00

*GFH* General Foot Health

Regarding test–retest reliability, all domains exhibited an ICC > 0.7, ranging from 0.75 to 0.80, with the exception of the Footwear domain, which exhibited an ICC of 0.69. The SEM_(agreement)_ ranged from 11.8 to 13.5, while the SDC_(individual)_ ranged from 32.7 to 37.4. Finally, the SDC_(group)_ was estimated to range from 5.1 to 5.8. Our data revealed a floor effect (> 15%) on the foot unction and GFH domains at baseline, as shown in Table 3.

**Table 3:** SEm, SDC and floor and ceiling effect

	Domains of foot-specific FHSQ (Questions covered)			
	Foot pain (1–4)	Foot function (5–8)	Footwear (10–12)	GFH (9 + 13)
SEm	12.3	13.5	11.8	13.0
SDC_Individual_	34.9	37.4	32.7	36.0
SDC_Group_	5.5	5.8	5.1	5.6
Floor effect (%)	6.9	22.2	8.6	21.8
Celling effect (%)	21.3	6.1	13.2	2.3

*FHSQ* Foot Health Status Questionnaire, *GFH* General Foot Health, *SEm* Standard Error of measurement, *SDC* Smallest detectable difference

All test done using 95% confidence interval

Also, only foot pain domain exhibited a ceiling effect that extended beyond the predefined 15%.

### Validity

As hypothesized, there was a significant correlation between mean heel pain and all the Arabic FHSQ domains, as shown in Table 4 foot pain and foot function domains have been estimated to have a moderate negative correlation (*r* =  − 0.73, *P* = 0.00, *r* =  − 0.71, *P* = 0.00). The footwear and GFH domains is estimated to have a poor negative correlation (*r* =  − 0.23, *P* = 0.04, *r* =  − 0.48, *P* = 0.00). Three out of four hypotheses were supported by the data.

**Table 4:** The outcomes of the evaluation conducted to test the construct validity of the Arabic FHSQ

	Domains of foot-specific FHSQ (Questions covered)			
	Foot pain (1–4)	Foot function (5–8)	Footwear (10–12)	GFH (9 + 13)
Spearman’s correlation coefficient FHSQ—VAS100mm (*n* = 77)	− 0.73	− 0.58	− 0.23	− 0.48
Confidence intervals	( − 0.84; − 0.55)	( − 0.72; − 0.40)	( − 0.42; − 0.02)	( − 0.66; − 0.27)
*P*-value	0.00	0.00	0.04	0.00
Predefined hypothesis	Moderate negative relationship	Moderate negative relationship	Poor negative relationship	poor negative relationship
Do the hypothesis proven?	Yes	Yes	Yes	No

*FHSQ* Foot Health Status Questionnaire, *GFH* General Foot Health, *VAS* Visual Analogue scale

All tests are significant at level < 0.05 (2-tailed)

## Discussion

A single-center investigation into the psychometric properties of the Arabic version of the FHSQ was conducted following its initial translation and cross-cultural adaptation for patients with PF. While validated Arabic tools exist for measuring foot function in certain diseases, such as the diabetic foot self-care questionnaire [32], the FHSQ score is unique in its ability to evaluate the effects of foot surgery, assess foot disorders, and provide an overall assessment of quality of life [9, 13]. As the best tool for assessing PF, it is crucial for patients to have access to a valid assessment tool across different cultures [14]. To facilitate this, the FHSQ should be available in multiple languages, including Arabic, to enable cross-cultural comparisons.

The translation adhered to the translation and back translation method [17, 18]. The overall structure and format of the questionnaire were preserved throughout all stages of the translation and adaptation process. Consensus regarding the appropriate translation of phrases such as "how often" was achieved during the initial translation steps. Following the completion of the translation process, a pre-test version of the Arabic FHSQ was administered to 30 patients. The initial evaluation phase indicated that patients had a comprehensive understanding of all questions. Feedback regarding potentially confusing Likert scales prompted their revision, and an additional 20 patients were included to verify the clarity of the newly edited Likert scales.

Among our population, foot function was the domain most severely impacted, with a score of 70.5, while physical activity was the least affected, with a score of 34.6. These results were lower than those observed in the general population of Saudi Arabia, as expected, and consistent with findings from a study conducted in a population of PF patients [33, 34]. These results are reflective of the urban population in Saudi Arabia, and it is anticipated that HrQoL may be lower among the rural population [35].

Following the completion of the cross-cultural adaptation process, we proceeded to evaluate the Arabic version of the FHSQ in our patient population. This paper included considerations such as dimensionality, internal consistency, reproducibility (encapsulating both reliability and agreement), interpretability, and construct validity. The principal component analysis (PCA) executed in our study brought forward a four-factor solution, accounting for a significant proportion of variance. Although this was somewhat inferior to the initial investigation [13], it rendered a satisfactory level of internal consistency as well as an indication of construct validity.

Internal consistency is a metric that quantifies the degree of correlation between items within a domain of a questionnaire, essentially signifying that they measure the same construct and exhibit homogeneity [25]. The original investigation reported internal consistency in the range of 0.851 to 0.884 [13]. However, the FHSQ-Ar demonstrated a Cronbach’s alpha > 0.90 across all domains, except the footwear domain, which still showcased a good internal consistency of 0.70.

The Arabic adaptation of the FHSQ exhibited satisfactory reproducibility, as presented through calculated reliability and agreement. The reproducibility of a test refers to how consistent the results are when the same test is repeated multiple times on the same individuals under stable conditions. Reliability was assessed using ICC_agreement_, with the footwear domain scoring the lowest (0.69) and the GFH domain scoring the highest (0.80). This concurs with the original authors' findings, where the footwear domain scored the lowest, a pattern that echoes in other transcultural adaptations of the FHSQ [13, 36].

Agreement was computed via the standard error of measurement (SEm) and the subsequent smallest detectable change (SDC) at both individual and group levels. Our analysis revealed SEm values ranging from 11.8 to 13.5 across the different FHSQ sub-scales, with footwear and foot function domains yielding the lowest and highest SEms, respectively. The resulting SDC_(individual)_ values for each domain varied from 32.7 to 37.4 at the individual level, implying that a change of at least 32.7 points in the footwear domain or 37.4 points in the foot function domain can be discerned as a true change rather than a measurement error. These results suggest that the translated and validated FHSQ may not be optimal for application at the individual level due to the high SDC values. However, SEm and SDC were not calculated in the original study for comparison, although they were computed in the Dutch transcultural adaptation of the FHSQ, which yielded analogous results [13, 36]. The calculated SDC_(group)_ in this study ranged between 5.1 in the footwear domain and 5.8 in the foot function domain, indicating that the FHSQ-Ar is suitable for conduction at the group level. The minimal detectable change (MID) of the FHSQ is estimated to vary from -0.3 to 13 across scales [37].

The validity of the four-factor model was corroborated by their associations with the VAS0-100 mm score. Three of four hypotheses (75%) were fulfilled. The footwear domain wasn’t anticipated to show a correlation since this domain gauges a construct hypothesized to be independent from heel pain intensity as per the original instrument [13]. The fit indices associated with the CFA models were satisfactory for the FHSQ-Ar.

Limitations of this study include reliance on an internet-based survey, curtailing the feasibility of retesting patients under identical circumstances—a less than ideal scenario for evaluating reliability and measurement error [38]. Furthermore, many respondents declined to be engaged in an online survey for a lack of trust, contributing to a response rate that was below the desired numbers. Additionally, our sampled population may not accurately reflect the demographic suffering from plantar fasciitis in Saudi Arabia, as there's an absence of comprehensive sociodemographic data for the region. The strengths of this study include the methodological assessments of the translation procedure and the inclusion of various psychometric properties aligned with standard guidelines. Adhering to these guidelines bolsters the credibility of our questionnaire's application. Lastly, though we've rendered the FHSQ into Arabic, its applicability needs further validation across other conditions, not limited to PF, in subsequent research.

## Conclusion

In conclusion, our study highlights the uneventful cross-cultural adaptation and validation of the FHSQ-Ar for use among patients with PF in Saudi Arabia. The FHSQ-Ar demonstrated good psychometric properties, including internal consistency, dimensionality, reproducibility, interpretability, and construct validity, comparable to the original and other adaptations of the FHSQ. Despite the high SDC values indicating possible limitations for individual patient assessments, the instrument proved viable for group-level assessments. The four-factor solution brought forward by PCA held satisfactory internal consistency and construct validity, further strengthening the FHSQ-Ar's credibility. Nevertheless, future research is needed to extend its validation across other conditions, not just PF (Additional file 1).

### Supplementary Information

**Additional file 1**. Arabic version of the FHSQ.

## Data Availability

The data sets that support the findings of this study are available from the corresponding author. Upon request, the data underlying the results presented in this study will be shared by the corresponding author.

## Appendix

See Tables 5 and 6.

**Table 5:** Transcultural adaptation of the Arabic Foot Health Status Questionnaire (FHSQ-Ar) with Likert scale adaptation

	Original FHSQ	FHSQ in Arabic
*Foot pain domain*		
	What level of foot pain have you had during the past week?	ما مستوى الألم خلال الأسبوع الماضي؟
	How often have you had foot pain?	هل تشعر عادةً بآلام القدم؟
	How often did your feet ache?	هل تشعر عادةً بآلام خفيفة متواصلة في قدمك؟
	How often did you get sharp pains in your feet?	هل تشعر عادةً بألم حاد في قدمك؟
Likert scale of Foot Pain domain		
Question 1	None; Very mild; Mild; moderate; Severe	لا يوجد، بسيط للغاية، بسيط، متوسط، شديد
Questions 2;3 and 4	Never; Occasionally; Fairly many times; Very ofter; Always	إطلاقًا، نادرًا، أحيانًا، غالبًا، دائمًا
*Foot function domain*		
	Have you feet caused you to have difficulties in your work or activities?	هل سببت لك قدمك صعوبات في أداء عملك أو نشاطاتك ؟
	Were you limited in the kind of work you could do because of your feet?	هل حدّت قدمك من أدائك لأعمال اعتدت القيام بها ؟
	How much does your foot health limit you walking?	إلى أي درجة حدّت صحة قدمك من مشيك؟
	How much does your health limit you climbing stairs?	إلى أي درجة حدّت صحة قدمك من استخدامك السلالم للصعود؟
Likert scale of Foot function domain		
Questions 5; 6; 7 and 8	Not at all; Slightly; Moderately; Quite a bit; Extremely	إطلاقًا، بعض الشيء نوعًا ما ، كثيرًا بشدة
*Footwear domain*		
	It is hard to find shoes that do not hurt my feet	من الصعب العثور على حذاء لا يؤذي قدمي
	I have difficulty in finding shoes that fit my feet	من الصعب العثور على حذاء يناسب قدمي
	I am limited in the number of shoes I can wear	أنا مقيد في عدد الأحذية التي يمكنني ارتدائها
Likert scale of Footwear domain		
Questions 10; 11 and 12	Strongly agree; Agree; Neither agree nor disagree; Disagree; Strongly disagree	أوافق بشدة، أوافق، محايد، لا أوافق، لا أوافق بشدة
*General Foot Health Domain*		
	How would you rate your overall foot health?	ما تقيمك لصحة قدمك بصورة عامة ؟
	In general, what condition would you say your feet are in?	بشكل عام، ما حال قدمك حاليًا؟
Likert scale of general foot health domain		
Question 9 and 13	Excellent; Very good; Good; Fair; Poor	ممتازة، جيدة جدًا، جيدة، مقبولة، سيئة
Likert scale of generic-FHSQ		
Question 14	Very good; Fair; Poor	جيدة جدًا، مقبولة، سيئة
Questions 15 (a–i)	Yew, limited a lot’; Yes, limited a little; no, not limited at all	نعم، تقيدني كثيرًا. نعم، تقيدني قليلًا. لا تقيدني إطلاقًا
Question 16	Not at all; Slightly; Moderately; Quite a bit; Extremely	إطلاقًا، بعض الشيء نوعًا ما ، كثيرًا بشدة
Questions 17 (a–d)	All of the time; Most of the time; Some of the time; Some of the times; A little of the time; None of the time	كل حين، معظم الأحيان، بعض الأحيان، أحيانًا قليلة، إطلاقًا
Question 18	No time at all; a small amount of time; Moderate amount of time; Quite a bit of the time; All of the time	إطلاقًا، بعض الوقت، أحيانًا، معظم الوقت، دائمًا
Questions 19 (a–d)	True or mostly true; Don’t know; False of mostly false	صحيحة، أو غالبًا صحيحة. لا أعلم. خاطئة أو غالبًا خاطئة

Questions 14 and later are part of the generic FHSQ, which was taken from the SF-36. Likert scales for questions 14 and 15 have been taken from the Arabic SF-36 health survey. Question 16, 17, 18, and 19, although being taken from the Arabic SF-36 health survey, have been changed to fit the FHSQ questionnaire

*FHSQ* Foot Health Status Questionnaire. *SF-36* 36-item short form survey

**Table 6:** Transcultural adaptation of the Arabic Foot Health Status Questionnaire (FHSQ-Ar) with Likert scale adaptation

Item (*N* = 87)	Value
Age in years ± SD	
Total	48.6 ± 10.1
Male	52.7 ± 14.1
Female	47.3 ± 7.7
Gender, *n* (%)	
Male	25 (27.5%)
Female	62 (68.1%)
Marital status, n (%)	
Married	69 (79.3%)
Single	18 (20.7%)
Educational level, n (%)	
≤ High school	10 (11.5%)
≥ University	77 (78.5%)
Occupational status, n (%)	
Not working	40 (46.0%)
Working with no prolonged standing (< 50% of a shift time)	24 (27.6%)
Working with prolonged standing (> 50% of a shift time)	23 (26.4%)
Exercise (hours a week), n (%)	
Not exercising at all	48 (55.2%)
Exercising, (less than 2.5)	14 (16.1%)
Exercising, (more than 2.5)	25 (28.7%)
*Prevalence of comorbidities among participants*	
Comorbodity, n (%)	
Diabetes mellitus	20 (22.9%)
Hypertension	18 (20.7%)
Thyroid diseases	5 (5.5%)
Heart diseases	1 (1.2%)
Kidney diseases	1 (1.2%)
Osteoporosis	1 (1.2%)
Osteoarthritis	1 (1.2%)

*SD* Standard deviation
