# Supplementary material for: Arabic translation, cross cultural adaptation, and validation of Foot Health Status Questionnaire among Saudi individuals with plantar fasciitis
Source: J Orthop Surg Res. 2023 Oct 4;18:754. doi: 10.1186/s13018-023-04202-9 (PMC10552317; doi:10.1186/s13018-023-04202-9)
Supplement: Supplementary file 1 — Additional file 1. Arabic version of the FHSQ. [file 13018_2023_4202_MOESM1_ESM.docx]

نموذج الموافقة المستنيرة لاجراء بحث طبى

عزيزي المريض..

- عنوان البحث: جودة الحياة في مرضى التهاب اللفافة الأخمصية في المملكة العربية السعودية
- عزيزي المشارك،

شكرًا على موافقتك على المشاركة في هذا الاستبيان الالكتروني لدراسة بعنوان: __________________.

- والغرض من هذا الاستبيان الالكتروني هو __________________ . وسيستغرق اكمال هذا الاستبيان حوالي _____.
- نؤكد لك أن جميع إجاباتك التي ستقدمها ستبقى في سرية تامة، لا تتردد في الاتصال بالأستاذ _________، رقم الهاتف المحمول _________. للإجابة على أسئلتك.

إذا كنت موافقًا على المشاركة في هذا الاستبيان الإلكتروني فيرجى النقر على "التالي" للبدء.

**الموقف من المشاركة في الاستبيان**

**أوافق على المشاركة في هذا الاستبيان طواعية**

**لا أوافق على المشاركة في هذا الاستبيان**

إذا كنت موافق على المشاركة في هذا الاستبيان الالكتروني فيرجى النقر على "التالي" للبدء.

فإذا كنت موافق على المشاركة برجاء التوقيع على الإقرار أدناه

ملحوظة: من حقك الحصول على صورة من الاقرار.

# القسم (أ): استبيان حالة صحة القدم:

ملاحظات:

1. أسئلة هذا الاستببيان تتمحور حول رؤيتك الشخصية لصحة قدمك
2. لتحديد إجابة، ضع دائرة على الإجابة التي تريدها لكل سؤال
3. إن لم تكن لديك إجابات لبعض الأسئلة، فحاول فضلًا أن تعطي الإجابة الأقرب.

| \| **الأسئلة التالية عن ألام القدم خلال الأسبوع الماضي:** \| \| --- \| | | | | |
| --- | --- | --- | --- | --- | --- |
| 1. ما مستوى الألم خلال الأسبوع الماضي: | | | | |
| - لا يوجد | - بسيط للغاية | - بسيط | - متوسط | - شديد |
| **خلال الأسبوع الماضي:** | | | | |
| 1. هل تشعر عادةً بآلام القدم؟ | | | | |
| - أطلاقًا | - نادرًا | - أحيانًا | - غالبًا | - دائمًا |
| 1. هل تشعر عادةً بآلام خفيفة متواصلة في قدمك؟ | | | | |
| - إطلاقًا | - نادرًا | - أحيانًا | - غالبًا | - دائمًا |
| 1. هل تشعر عادةً بألم حاد في قدمك؟ | | | | |
| - إطلاقًا | - نادرًا | - أحيانًا | - غالبًا | - دائمًا |
| \| **الأسئلة التالية عن مدى تأثر روتينك اليومي بحالة قدمك:** \| \| --- \| | | | | |
| **خلال الأسبوع الماضي:** | | | | |
| \| 1. هل سببت لك قدمك صعوبات في أداء عملك أو نشاطاتك ؟ \| \| \| \| \| \| --- \| --- \| --- \| --- \| --- \| \| - إطلاقًا \| - بعض الشيء \| - نوعًا ما \| - كثيرًا \| - بشدة \| \| 1. هل حدّت قدمك من أدائك لأعمال اعتدت القيام بها ؟ \| \| \| \| \| \| - إطلاقًا \| - بعض الشيء \| - نوعًا ما \| - كثيرًا \| - بشدة \| \| **خلال الأسبوع الماضي:** \| \| \| \| \| \| 1. إلى أي درجة حدّت صحة قدمك من مشيك؟ \| \| \| \| \| \| - إطلاقًا \| - بعض الشيء \| - نوعًا ما \| - كثيرًا \| - بشدة \| \| 1. إلى أي درجة حدّت صحة قدمك من استخدامك السلالم للصعود؟ \| \| \| \| \| \| - إطلاقًا \| - بعض الشيء \| - نوعًا ما \| - كثيرًا \| - بشدة \| \| 1. **ما تقيمك لصحة قدمك بصورة عامة ؟** \| \| \| \| \| \| - ممتازة \| - جيدة جدًا \| - جيدة \| - مقبولة \| - سيئة \| | | | | |

| \| **الأسئلة التالية تتعلق بالحذاء الذي ترتديه. يرجى اختيار الخيار الذي يصف موقفك على أفضل وجه** \| \| --- \| | | | | | | |
| --- | --- | --- | --- | --- | --- | --- | --- |
| 1. من الصعب العثور على حذاء لا يؤذي قدمي | | | | | | |
| - أوافق بشدة | - أوافق | - محايد | | - لا أوافق | - لا أوافق بشدة | |
| 1. من الصعب العثور على حذاء يناسب قدمي | | | | | | |
| - أوافق بشدة | - أوافق | - محايد | | - لا أوافق | - لا أوافق بشدة | |
| 1. أنا مقيد في عدد الأحذية التي يمكنني ارتدائها | | | | | | |
| - أوافق بشدة | - أوافق | - محايد | | - لا أوافق | - لا أوافق بشدة | |
| 1. **بشكل عام، ما حال قدمك حاليًا؟** | | | | | | |
| - ممتازة | - جيدة جدًا | - جيدة | | - مقبولة | - سيئة | |
| **فضلا.. اكتب بعض التعليقات عن صحتك قدمك الحالية:**  **.................................................................................................................................... .................................................................................................................................... ....................................................................................................................................** | | | | | | |
| 1. **ما تقييمك لحالتك الصحية بشكل عام:** | | | | | | |
| - جيدة جدًا | | | - مقبولة | | | - سيئة |
| \| 1. **تتعلق البنود التالية بأنشطة يمكن أن تقوم بها خلال يومك العادي.**   **في الوقت الحالي، إلى أي مدى تقيدك حالتك الصحية:** \| \| --- \| | | | | | | |

| لا تقيدني إطلاقًا | نعم، تقيدني قليلًا | نعم، تقيدني كثيرًا | النشاطات |
| --- | --- | --- | --- |
| 3 | 2 | 1 | من ممارسة الأنشطة الشاقة مثل الجري، حمل الأشياء الثقيلة أو مزاولة الأنشطة الرياضية المجهدة جدًا |
| 3 | 2 | 1 | من ممارسة الأنشطة متوسطة الجهد كتحريك الطاولة أو التنظيف باستخدام المكنسة الكهربائية أو تنظيف حديقة المنزل والعناية بها؟ |
| 3 | 2 | 1 | من حمل المشتريات من البقالة أو السوق المركزي (آلسوبرماركت)؟ |
| 3 | 2 | 1 | من صعود الدرج لعدة أدوار |
| 3 | 2 | 1 | من صعود الدرج لدور واحد فقط؟ |
| 3 | 2 | 1 | من الانحناء أو الركوع أو السجود ؟ |
| 3 | 2 | 1 | من المشي لمسافة أكثر من 1 كيلومتر؟ |
| 3 | 2 | 1 | المشي لمسافة 100 متر |
| 3 | 2 | 1 | من الاستحمام أو ارتداء ملابسك بنفسك |

| 1. خلال الأسابيع الأربعة الماضية، هل تعارضت صحتك الجسمية او النفسية مع تأديتك لنشاطاتك الاجتماعية المعتادة مع عائلتك او اصدقائك او جيرانك او أي من المناسبات الاجتماعية الأخرى؟ | | | | |
| --- | --- | --- | --- | --- |
| - إطلاقًا | - بعض الشيء | - نوعًا ما | - كثيرًا | - بشدة |
| 1. **الأسئلة التالية تتعلق بكيفية شعورك وطبيعة سير الأمور معك خلال الأسابيع الأربعة الماضية، الرجاء إعطاء إجابة واحدة لكل سؤال بحيث تكون هذه الإجابة هي الأقرب الى الحالة التي كنت تشعر بها. خلال الأسابيع الأربعة الماضية، كم من الوقت:** | | | | |

| إطلاقًا | أحيانًا قليلة | بعض الأحيان | معظم الأحيان | كل حين |  |
| --- | --- | --- | --- | --- | --- |
| 5 | 4 | 3 | 2 | 1 | شعرت بأنك تعبان؟ |
| 5 | 4 | 3 | 2 | 1 | شعرت بانك مليء بالحيوية والنشاط؟ |
| 5 | 4 | 3 | 2 | 1 | شعرت بأنك منهك (استُنْفِذت قواك)؟ |
| 5 | 4 | 3 | 2 | 1 | كانت لديك طاقة كبيرة؟ |

| 1. خلال الأسابيع الأربعة الماضية، ما مقدار الوقت الذي تعارضت فيه صحتك الجسمية او مشاكلك النفسية مع نشاطاتك الاجتماعية (مثل زيارة الأصدقاء والأقارب وغير ذلك)؟ | | | | |
| --- | --- | --- | --- | --- |
| - إطلاقًا | - بعض الوقت | - أحيانًا | - معظم الوقت | - دائمًا |
| 1. ما مدى **صحة** أو **خطأ** كل من العبارات التالية (أ،ب،ت،ث) بالنسبة الى حالتك الصحية؟ | | | | |

| خاطئة، أو غالبًا خاطئة | لا أعلم | صحيحة، أو غالبًا صحيحة | النشاطات |
| --- | --- | --- | --- |
| 3 | 2 | 1 | يبدو أنني أصاب بالمرض أسهل من الآخرين. |
| 3 | 2 | 1 | حالتي الصحية مساوية لأي شخص أعرفه. |
| 3 | 2 | 1 | أتوقع أن تسوء حالتي الصحية. |
| 3 | 2 | 1 | حالتي الصحية ممتازة. |

# القسم (): بيانات المريض ومعلومات عن المرض:

| 1. **الاسم كاملًا:** | **................................................................** | | | |  |
| --- | --- | --- | --- | --- | --- |
| 1. **العنوان** | **............................................................... رمز بريدي: ...........** | | | | |
| 1. **الجنس** | - ذكر | - أنثى | |  |  |
| 1. **تاريخ الميلاد** | **................................................................** | | | | |
| 1. **تاريخ تعبئتك لهذا الاستبيان** | **................................................................** | | | | |
| 1. **هل تأخذ حاليًا أية أدوية موصوفة من طبيبك لأي من الأمراض الآتية:** | - داء السكري | | - خشونة المفاصل | - ارتفاع ضغط الدم | - أمراض القلب |
|  | - أمراض الرئة | | - علاج هرموني تعويضي | - الروماتيزم | - آلام الظهر |
|  | - الاكتئاب | | - أخرى، فضلًا عدد: **............................................** | | |
| \| **بخصوص الأسئلة التالية، فضلًا حدد الإجابة أما نعم أو لا** \| \| --- \| | | | | | |

1. **هل أنت من مستفيدي الضمان؟**

| - نعم | - لا |
| --- | --- |

1. **هل تدخن ؟**

| - نعم | - لا |
| --- | --- |

1. **هل تمارس أية نوع من الرياضة؟**

| - نعم | - لا |
| --- | --- |

1. **هل لديك تأمين صحي خاص ؟**

| - نعم | - لا |
| --- | --- |

1. **هل أكملت أية شهادة عمل أو تدريب أو أية كفاءات تعليمية من بعد أن أكملت مراحلك الدراسية ؟**

| - نعم | - لا |
| --- | --- |
